# Medical Opinion and Movement

**Published:** 1907-03-09

**Authors:** 


### Medical Opinion and Movement.

An interesting discussion took place at a meeting of the Royal Medical and Chirurgical Society on February 26 on Human and Bovine Tuberculosis. Dr. Nathan Row sought to establish his belief that the two kinds of infection of the human subject could be definitely distinguished, that the typus humanus gave rise to phthisis pulmonalis, tuberculous laryngitis, and secondary ulceration of the intestines, whereas other forms of the disease, including acute miliary tuberculosis, were due to the bovine type. He advanced evidence from inoculation experiments on animals to show that tuberculous glands of the neck were of bovine origin, resulting from absorption from the mouth and tonsils. He suggested, further, that human and bovine tuberculosis were antagonistic to one another, and that a mild attack of one conferred immunity against the other. These views are contrary to the findings of the Royal Commission, which was unable to make any such distinction between cases due to infection from one or other source. During the discussion Dr. Lawson pointed out that it was not uncommon for a case of phthisis pulmonalis to terminate in acute miliary tuberculosis. Dr. F. M. Sandwith made some interesting observations on tuberculosis in Egypt. Every form of tuberculosis, except meningitis, is common in that part, although tubercle in cattle is extremely rare. Moreover, he stated that the mothers often suckle their children for two years, and when cow's milk was given it was nearly always boiled. He suggested that the investigation of the disease in Egypt might help to elucidate this much vexed question of the relation of the two types of disease.

Dr. Lauriston E. Shaw chose a somewhat unusual theme for his Hunterian oration, but one full of interest and importance. From the suggestion that Hunter's education probably gave his genius a freer scope for development, Dr. Shaw proceeds to discuss the evils of what he calls the examination fetish of modern medical education. Dr. Shaw was for several years Dean to Guy's Hospital Medical School, and is still a teacher of medicine in the same school and a member of the Senate of the London University, so that his views on medical education are based upon considerable practical experience, and are entitled to consideration and respect. Dr. Shaw condemns the examination system as a "blighting influence upon the whole work of teachers and pupils from beginning to end of the modern medical curriculum." He points out that the examiner, who knows nothing of the student apart from his "number," can only test and ascertain the facts crammed into his brain, but can draw no conclusions in regard to his mental powers. In consequence it is examination facts that the student craves and demands from his teachers, not medical education in the proper sense of the term. Dr. Shaw would like to see the so-called "external" examinations replaced by "internal" examinations, in which the teacher also takes a share in testing the ability of his pupil. This would be rendered more possible if one could eliminate the danger of competition between the schools for students by a downward grade of examination standards. This can only be attained by placing the schools on a proper financial basis, so that the teachers are independent of the annual entries of students, and could be trusted to "brand their own herrings." But in the meantime much might be done to increase the value of practical work in the eyes of the students and secure its recognition by examination authorities by a system of inspection, and Dr. Shaw holds out some hope that the University of London may see its way to adopting some such scheme to modify the evils of the present examination system.

In a recent issue we dealt with the report of Dr. W. J. Morton, of New York, on the cases of cancer treated by him with injections of trypsin and amylopsin. The report showed little to justify the outbursts of enthusiasm indulged in by Dr. Saleeby in the lay Press previous to its appearance. Great importance, however, was attached to one case, in which the patient, after a trial of the treatment, had submitted to operation, and a pathological examination of the tumour removed showed evidence of degenerative changes taking place in the cancer-cells. From this observation Dr. Morton arrived at the rather hasty conclusion that the trypsin treatment had set up a process of degeneration and atrophy, which, if continued, would have resulted in the entire disappearance of the tumour. We now have an account of the case by the surgeon, Dr. Bainbridge, who performed the operation, and it throws rather a sinister light upon the mental attitude of the investigator, who makes deductions in harmony with his own preconceived ideas rather than in accordance with the facts of the case. It now appears that the pathological observations referred to were made on a small section of the tumour near the nipple, taken by Dr. Morton at the time of operation. The rest of the tumour was, however, submitted to examination by three leading pathologists, and metastatic growths which were removed at a subsequent operation were also sent to several pathologists to report upon. These reports are now published by Dr. Bainbridge, and they show that the conclusion of Dr. Morton that the growth was on the way to complete degeneration and atrophy was wholly unjustifiable. Degenerative changes are noted in the reports, but at the same time most of the sections showed signs of active growth, and the tumour presented in many sections the typical appearance of an actively growing scirrhus of the breast. Dr. Bainbridge, in making this report, in no way condemns the trypsin treatment as "tried and found wanting," but begs for "suspended judgment" and for "the cultivation of that enthusiasm for truth which should be brought to bear on all questions involving human life."

